# Development of a tool to assess HIV prevention readiness of adolescent girls and young women in HPTN 082 study

**DOI:** 10.1371/journal.pone.0281728

**Published:** 2023-02-24

**Authors:** Geetha Beauchamp, Sybil Hosek, Deborah J. Donnell, Kwun C. G. Chan, Brian P. Flaherty, Peter L. Anderson, Bonnie J. Dye, Nyaradzo Mgodi, Linda-Gail Bekker, Sinead Delany-Moretlwe, Connie Celum, for the HPTN 082 study team

**Affiliations:** 1 Department of Health Systems and Population Health, University of Washington, Seattle, Washington, United States of America; 2 Vaccine and Infectious Disease Division, Fred Hutchinson Cancer Research Center, Seattle, Washington, United States of America; 3 Department of Psychiatry, Stroger Hospital of Cook County, Chicago, Illinois, United States of America; 4 Department of Biostatistics, University of Washington, Seattle, Washington, United States of America; 5 Department of Psychology, University of Washington, Seattle, Washington, United States of America; 6 Department of Pharmaceutical Sciences, University of Colorado-Anschutz Medical Campus, Aurora, Colorado, United States of America; 7 FHI 360, Durham, North Carolina, United States of America; 8 University of Zimbabwe Clinical Trials Research Centre, Harare, Zimbabwe; 9 The Desmond Tutu HIV Centre, University of Cape Town, Cape Town, South Africa; 10 Wits RHI, Faculty of Health Sciences, University of the Witwatersrand, Johannesburg, South Africa; 11 Departments of Global Health, Medicine, and Epidemiology, University of Washington, Seattle, Washington, United States of America; University of Ottawa, CANADA

## Abstract

**Background:**

African adolescent girls and young women (AGYW) represent a large proportion of new HIV infections, a priority population for pre-exposure prophylaxis (PrEP), but adherence remains a challenge. A reliable, valid readiness tool would help identify AGYW motivated to take PrEP who need adherence support.

**Methods:**

In the HPTN 082 open-label PrEP study (2016–2019), South African and Zimbabwean women ages 16–25 were administered an HIV prevention readiness measure (HPRM). The 25 items in the HPRM included medication beliefs, connection with care, disclosure of PrEP use, social support, and housing stability using a 5-point Likert scale. Exploratory factor analysis (EFA) using polychoric correlations, scale reliability, and predictive validity were performed on data from 315 participants who responded to all items. We assessed the predictive value of HPRM scores with PrEP adherence, defined as tenofovir-diphosphate (TFV-DP) concentrations in dried blood spots, as a continuous measure and dichotomized as high PrEP adherence (≥700 fmol/punch).

**Results:**

EFA yielded 23 items with three subscales: self-efficacy (16 items), PrEP disclosure (4 items), and social support (3 items). Cronbach’s α ranged from 0.71 to 0.92 for the overall scale and the subscales. The average overall scale and the subscales were predictive of 3-month PrEP adherence for TFV-DP concentrations: for each unit increase of the HPRM score, TFV-DP concentration increased by 103 fmol/punch (95% CI: 16, 189, *p* = 0.02); the highest HPRM score equated with 608 fmol/punch on average. For the self-efficacy subscale, TFV-DP increased by 90 fmol/punch (95% CI: 7, 172, *p* = 0.03); PrEP disclosure, 68 fmol/punch (95% CI: 19, 117 p = 0.01); and social support, 58fmol/punch (95% CI: 2, 113, *p* = 0.04). Higher PrEP disclosure suggests high adherence (OR 1.36, 95% CI: 1.00, 1.86, *p* = 0.05) and predicted persistent high adherence at both months three and six (OR: 1.50, 95% CI: 1.03, 2.21, *p* = 0.04).

**Conclusions:**

The HPRM scale overall and the subscales individually demonstrated good internal consistency among African young women. PrEP disclosure subscale exhibiting significant association with persistent high PrEP adherence is an important finding for PrEP adherence support programs. Future work will assess replicability and expand self-efficacy and social-support subscales after item revision.

**Trial registration:**

ClinicalTrials.gov NCT02732730.

## Introduction

Even with increased HIV testing and access to treatment for persons living with HIV, in 2019, adolescent girls and young women (AGYW) in sub-Saharan Africa accounted for the most substantial (25%) proportion of 1.7 million new HIV infections worldwide [1]. Oral pre-exposure prophylaxis (PrEP) is highly effective in preventing HIV if medication is taken consistently [2–5]. However, taking a daily oral pill to prevent future infection is challenging, especially for African AGYW, a priority population given persistently high HIV incidence rates [6, 7]. In several open-label PrEP demonstration studies among AGYW, proportions of high PrEP adherence were between 25%- 60% within the first six months [8–10]. For adherence interventions to be effective, intervention support needs to be tailored to address the barriers unique to AGYW.

Qualitative studies among women in Africa indicate that the reasons for not consistently taking PrEP are multifaceted, including depression, intimate partner violence, fear of disclosure of PrEP use, stigma, low community awareness of the effectiveness of PrEP, and medication and institutional distrust [11–14] Adherence behavior is not simply a personal decision; environmental ‘push’ and ‘pull’ factors affect young woman’s ability to take PrEP pills consistently. The socio-ecological model (SEM) framework is helpful for considering facilitators and barriers to adherence that include individual (e.g., psychological issues, medication beliefs, discomfort with disclosure), intrapersonal (e.g., social support, intimate partner violence), institutional (e.g., provider characteristics, navigating complex health systems), and structural (e.g., housing, food insecurities) factors [15]. These multifaceted barriers likely complicate AGYW’s ability to recognize obstacles and communicate to her provider effectively. Thereby, providers are impeded from offering adherence support that may mitigate obstacles to HIV prevention. In addition, it is difficult for the providers to adequately address barriers (e.g., food scarcity) due to the largeness of the problem [16]. Given the complexity of factors influencing PrEP adherence, a reliable, valid, comprehensive low-cost tool to assess the prevention readiness of AGYW is necessary and would be valuable to HIV prevention providers and researchers in sub-Saharan Africa [17].

**Fig 1:**
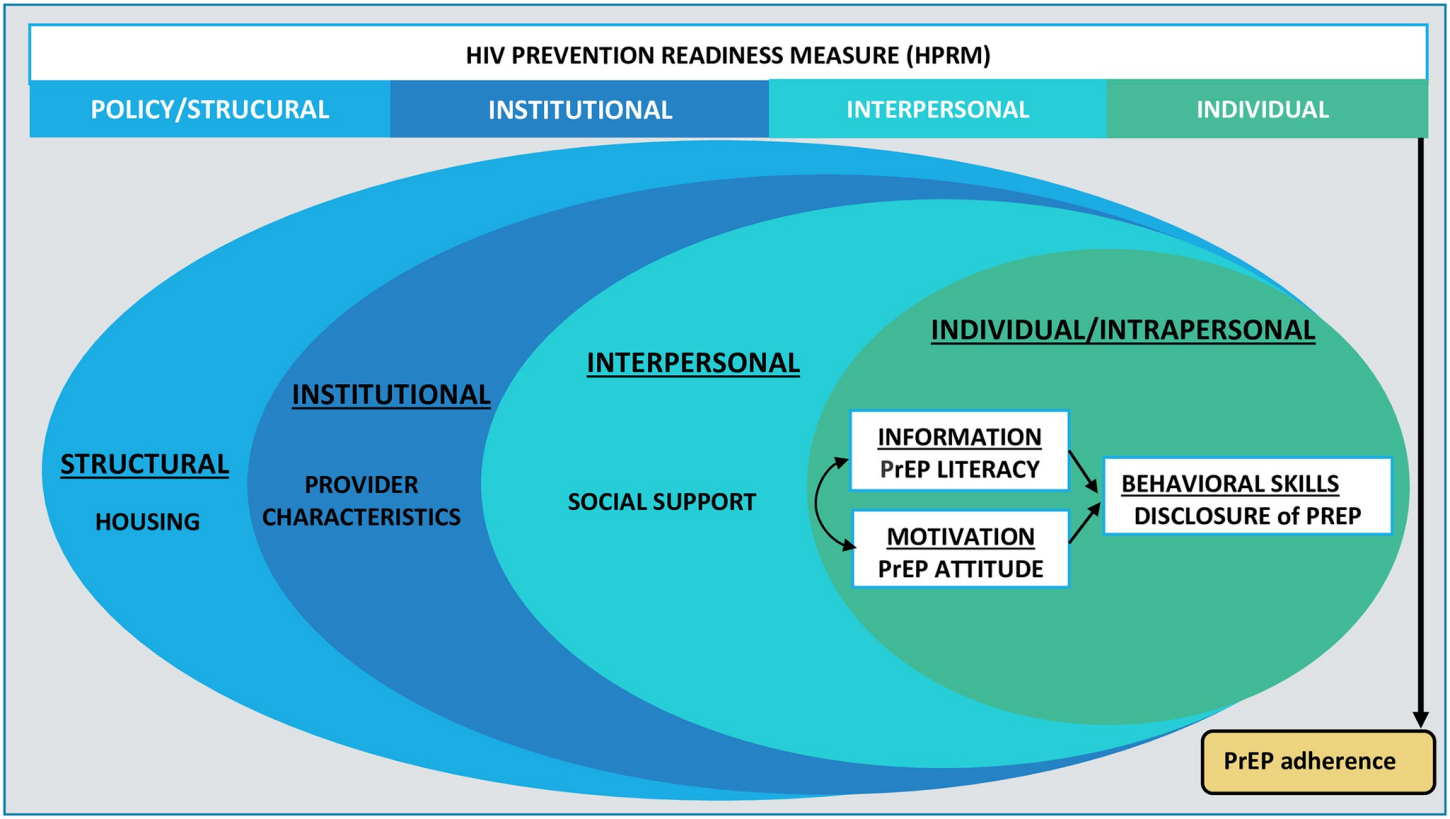
Mapping of HIV prevention readiness measure to situated-Information, Motivation and Behavioral framework (sIMB).

To assess HIV prevention readiness, we adapted a validated HIV *treatment* readiness measure (HTRM) developed for youth (ages 13–24 years) living with HIV infection in the U.S. [18]. The HTRM components encompassed several socio-ecological barriers, including disclosing HIV infection status to friends and family, psychological issues, connection-with-care, HIV medication beliefs, and alcohol and drug use. The overall HTRM scale and the subscales (i.e., constructs) medication belief, psychological issues, and connection-with-care demonstrated high internal consistency and predicted future viral suppression [19]. The HIV *prevention* readiness (HPRM) framework conceptualized core determinants of PrEP adherence behavior through the lens of situated-Information, Motivation and Behavioral model (sIMB) that Amico proposed in 2011 [20]. The sIMB model acknowledges that individuals are situated in a larger ecosystem that affects their ability to adhere to the recommended medical care for chronic conditions (e.g., HIV infection) [21]. The sIMB model combined two well-recognized theoretical models: the Information, Motivation and Behavioral (IMB) model [22, 23] with the socio-ecological model (SEM) [24]. Thus, our study leverages sIMB’s framework to contextualize that an individual’s HIV prevention behavior is affected by interpersonal relationships and institutional and structural factors, which augment or attenuate a young woman’s ability to adhere to PrEP as prescribed (Fig 1). This analysis evaluated the psychometric properties and predictive validity of HPRM items administered to AGYW in Zimbabwe and South Africa. We hypothesized that higher HPRM scale scores were likely associated with a greater likelihood of adherence to PrEP.

## Methods

### Ethical review

The HPTN 082 parent study received institutional review boards (IRB) approval from each study site: University of Cape Town Faculty of Health Sciences (reference number: 129/2016), University of Witwatersrand, Human Research Ethics Committee (reference number: 160304), and University of Zimbabwe Joint Research Ethics Committee (reference Number: 27/16). This work was supported by award numbers UM1AI068619, UM1AI068613, and UM1AI1068617 from the NIH (National Institute of Allergy and Infectious Diseases [NIAID]) to HPTN. All participants provided written informed consent in English or their local language. Following local regulations, participants below the legal age for consent provided assent, and parent or guardian informed consent was obtained.

### Study design overview

Study population, intervention, and laboratory procedures have been previously described in detail elsewhere [10]. Briefly, HPTN 082, an open-label PrEP demonstration study, enrolled 451 AGYW between the ages of 16 to 25 years old from youth-friendly clinics in Harare, Zimbabwe, and Cape Town, and Johannesburg, South Africa. The AGYW were recruited over two years between 2016 and 2018 and were followed for one year with visits at one and two months, and then quarterly. All participants were offered daily oral PrEP at enrollment and had the option to start PrEP until week 48 after enrollment. Participants could stop and restart PrEP with the clinician’s guidance at any point during the study or stop PrEP permanently. The protocol specified PrEP discontinuation reasons included HIV infection, Grade 3 toxicity related to PrEP pills, and pregnancy. The HPTN 082 study demonstrated high PrEP initiation (95%) among young African women. However, high PrEP adherence, defined as tenofovir-diphosphate (TFV-DP) ≥ 700 fmol/punch, was low (21%) at month six.

### Measures

#### HIV prevention readiness measure (HPRM)

The HPRM scale, consisting of 25 items (S1 Appendix), was administered at enrollment, and was repeated at month three for those who initiated PrEP. The content of the 25-items included were components of connection with care (5 items), medication beliefs (11 items), disclosure (4 items), and support (housing, general and PrEP support, 5 items) (Table 1). Responses were on a 5-point Likert scale ranging (0 to 4) from "strongly disagree", "disagree", "neither agree to disagree", "agree", to "strongly agree". The response for “How many people that you live with will you tell that you are taking PrEP?" was also on a 5-point Likert scale from "no one, "only one person", "some people", "most people" to "everyone". The HPTN 082 investigators modified some items to reflect three months of PrEP use when HPRM was repeated three months after starting PrEP. For example, "I think my household members who I will tell that I am on PrEP will help me remember to take it" was modified to "My household members who know I am on PrEP help me remember to take it". One of the original developers of the HTRM questionnaire [18, 19], who is a co-author of this paper, modified the questions to use in the HPRM (S1 Appendix) and granted permission to use the data for this manuscript.

**Table 1:** HPRM components and exploratory factor analysis loadings.

Item	HPRM Component	HPRM Items	Self-efficacy	PrEP disclosure	Social support
1	**Medication beliefs**	I am ready to take PrEP to protect against HIV.	0.514		
2		I believe taking PrEP can keep me from getting HIV.	0.760		
3		Even if I had side effects, I would take my PrEP because I know they would go away.	0.362		
4		I think that taking PrEP would give me side effects.^(R)^	Item Dropped		
5		If I don’t take my PrEP exactly as instructed, I might get infected with HIV.	0.547		
6		I know that I will be able to take my PrEP daily.	0.617		
7		Taking my PrEP as prescribed would keep me from getting HIV.	0.582		
8		It would be important to me to take my PrEP as instructed.	0.700		
9		I think taking PrEP would not really help me.^(R)^	0.316		
10		I think PrEP would be harmful to my body.^(R)^	Item Dropped		
11		I want to start taking PrEP to protect against HIV infection.	0.801		
12	**Connection with Care**	I have a strong, trusting relationship with the study staff.	0.424		
13		Even when it may be difficult, I will be able to let the study staff know if I miss doses of my PrEP.	0.707		
14		I regularly go to a clinic to seek advice about my health.	0.436		
15		I would know how to contact the study doctor/nurse if I had problems or questions about PrEP.	0.786		
16		I would know who to call and where to go for refills of my PrEP.	0.632		
17	**Disclosure**	I will tell most of the people that I live with that I am taking PrEP.		0.866	
18		I will tell most of my family and friends that I am taking PrEP.		0.759	
19		How many people that you live with will you tell that you are taking PrEP?		0.694	
20		I think I will not tell the people I live with that I am taking PrEP.^(R)^		0.502	
21	**Support**	I feel supported by my family and friends when times are tough.			0.694
22		I think my family and friends who I will tell I am on PrEP would help me remember to take it.			0.955
23		I think my household members who I will tell that I am on PrEP will help me remember to take it.			0.712
24		I feel like I have a stable pace to live.	0.785		
25		Sometimes I don’t have a place to sleep.^(R)^	0.505		

^(R)^ Reverse-coded. Self-efficacy (items 1–3, 5–9, 11–16, 24, and 25); PrEP disclosure (items 17–20); and Social support (items 21–23). Items 4 and 10 were dropped based on weak factor loadings.

Demographic data were collected at enrollment via case report forms (CRF). Patient-reported data, including HPRM items and other behavioral data, were collected using computed assisted self-interview (CASI). The question ’How would you describe your chances of getting HIV in the next year’ was used to assess the perception of HIV risk, which was collected using CASI.

#### PrEP adherence and persistent adherence

We evaluated the daily dosing of PrEP adherence using tenofovir-diphosphate (TFV-DP) concentration in dried blood spots (DBS). Tenofovir has a half-life of 17 days, and intracellular levels of the metabolite, tenofovir-diphosphate, accumulate in blood cells in a dose-proportional manner and are considered a biomarker of cumulative adherence for the prior 4–6 weeks [25]. An effectiveness threshold of DBS TFV-DP concentration to prevent HIV infection is not yet established for women. Therefore, we used the threshold of TFV-DP ≥ 700 fmol/punch as a biomarker of high adherence, a threshold established for men who have sex with men, where a 96% HIV risk reduction was associated with ≥ 4 doses per week corresponding to DBS TFV-DP ≥ 700 fmol/punch among MSM [26, 27]. A participant is considered ‘non-adherent’ to the prescribed daily dosing if the TFV-DP < 700 fmol/punch.

The predictive validity of the overall HPRM scale and subscales for adherence to PrEP were assessed using DBS TFV-DP concentration in three ways: 1) continuous measure at month three; 2) high adherence (dichotomized at TFV-DP ≥ 700 fmol/punch) at month three; and 3) persistent high adherence at both months three and six. High adherence only at one of the visits or neither visit was defined as not persistently dosing at high levels between months three and six. If PrEP adherence was missing for one of the visits, then it was set to missing.

### Statistical analysis

We conducted exploratory factor analysis (EFA) using complete cases to identify latent constructs that represent different aspects associated with PrEP adherence. Since the item responses were on an ordinal scale, we used polychoric correlations [28]. We expected some items to load on more than one factor (also referred to as subscale or latent construct) due to interrelation among items. Hence, we used oblique rotation (promax) and orthogonal rotation (varimax) to verify that the overall interpretation of the constructs was consistent. The scree plot combined with factor loading > 0.3 was used to determine the parsimonious interpretable number of factors that explained the highest proportion of variance [28, 29]. The difference > 0.2 criteria were applied to retain the item on the factor with the highest loading for items that loaded into multiple factors [30]. Further, the items with factor loadings < 0.3 were dropped in the final EFA. In sensitivity analysis, we performed EFA using the two-stage maximum likelihood (TSML) method to assess the impact of missing values, which can handle incomplete non-normally distributed data [31]. Reliability was assessed using Cronbach’s alpha coefficient to examine internal consistency; scales with Cronbach’s alpha coefficients > 0.7 are considered to have good scale reliability [32].

Before assessing predictive validity, the five negatively worded items (Table 1: items 4, 9, 10, 20, and 25) were reverse-coded to be consistent with positively framed items. Predictive validity was conducted in three ways. First, logistic regression was used for the binary outcome, high PrEP adherence at month three, by regressing on average overall HPRM scores and average subscales’ scores. Second, linear regression assessed the predictive validity of HPRM scores using month three TFV-DP as a continuous measure of PrEP adherence. TFV-DP concentrations that were below detection were set to half of the lowest detectable value at 16.55 fmol/punch. Lastly, logistic regression was used to evaluate the association between HPRM (overall scores and subscales’ scores) with persistent high adherence from months three to six. Linear regression was used to assess the association between HIV risk perception and HPRM. Logistic regression and linear regression models included the recruitment site as a covariate. Intraclass correlation was used to assess the temporal stability using overall and subscales’ scores of HPRM administered before and after starting PrEP (29). Except for TSML sensitivity analysis (R version 4.0.4), all analyses were conducted using SAS version 9.4 (SAS Institute, Cary, NC). The HPTN 082 trial is registered with ClinicalTrials.gov, NCT02732730.

## Results

### Participant characteristics

General characteristics are summarized for the 371 AGYW who completed the month three visit after starting PrEP medication and provided DBS samples. At enrollment, the median age of this cohort was 21 (IQR: 19 to 22, minimum to maximum: 16 to 26), 59% completed secondary education, and 89% had a regular place to stay. The majority (87%) reported a primary partner and having condomless vaginal sex (83%) in the past month. Half of the AGYW had a primary partner living with HIV infection or whose HIV status was unknown (51%) and perceived at least a slight chance of getting HIV in the next year (53%). They also reported some form of intimate partner violence in the past year (47%), and reported depression symptoms (49%) (Table 2).

**Table 2:** Baseline characteristics of the participants by month three adherence.

Baseline Characteristic N (%) or Median (IQR)	Overall N = 371	Adherent N = 92	Non-adherent N = 279
**Age (years)**	21 (19,22)	21 (20,22)	21 (19,22)
**Secondary education completed or higher**	219 (59%)	54 (59%)	165 (59%)
**Regular place to stay**	332 (89%)	84 (91%)	248 (89%)
**At least a small chance of getting HIV, next year** ^a^	197 (53%)	58 (63%)	139 (50%)
**Any alcoholic drinks, last 3 months**	256 (69%)	67 (73%)	189 (63%)
**Has Primary Partner, last 3 months**	313 (87%)	84 (91%)	229 (82%)
**HIV positive or unknown primary partner**	190 (51%)	43 (47%)	147 (53%)
**Age difference with primary partner, median (years)**	5 (2,7)	5 (3,7)	4 (2,6)
**Condomless vaginal sex, last month** ^b^	225/287 (78%)	64/78 (82%)	161/209 (77%)
**Condomless anal sex, last month** ^b^	43/257 (17%)	7/71 (10%)	36/186 (19%)
**Have more than one sex partner, last 3 months**	50 (13%)	6 (7%)	44 (16%)
**Had alcohol before sex, last month**	100 (27%)	23 (25%)	77 (28%)
**Transactional sex, last 3 months** ^c^	88 (24%)	24 (26%)	64 (23%)
**Sexually transmitted disease** ^d^	145 (39%)	37 (40%)	108 (39%)
**CES-D Depression score** **≥** **10** ^e^	183 (49%)	39 (42%)	144 (52%)
**Intimate partner violence in the past year** ^f^	175 (47%)	45 (49%)	130 (47%)

^a^ ‘How would you describe your chances of getting HIV in the next year’ was dichotomized as *at least a small chance* (small/ moderate, great chance and prefer not to answer) and *none* (no risk).

^b^ Amongst women who responded.

^c^ transaction sex is defined as having sex with a man in exchange for food, clothes, cosmetics, transportation, and items for children, and other items.

^d^ Positive test results for trichomonas, chlamydia, or gonorrhea.

^e^ Center for Epidemiologic studies Depression scale is the sum of 10 items (CESD-10) with a range of 0 to 30. CESD-10 score ≥ 10 indicates depressive symptoms.

^f^ Intimate partner violence is defined as having experienced emotional, physical, sexual violence and/or feeling unsafe from a sexual partner.

### Psychometric properties of the HPRM

The HPRM scale with 25 items were mapped to corresponding HPRM components (Table 1). Examination of the scree plot suggested a three-factor solution. Two of the five reverse-coded items (items 4 and 10) which related to whether AGYW viewed PrEP as harmful to her body, were dropped due to low loadings < 0.3 on all three factors. They also exhibited weak correlation with other items. Reverse-coded items 9 and 24 loaded into Factor 1, and item 20 loaded into factor 2. The 3-factor solution explained 46% of the variance of the items and was selected as parsimonious and interpretable. We examined the items that loaded on multiple factors and assigned them to the factor with the largest loading and best interpretability. Factor 1, labeled as ‘self-efficacy,’ consisted of items related to an individual’s beliefs about PrEP, including PrEP efficacy, ability to take PrEP as instructed, relationship with the providers to resolve medical issues, and having a stable place to live/sleep. Factor 2 was related to disclosure of PrEP use to family/friends and household members, and thus it was labeled as ‘PrEP disclosure’. Factor 3 was related to general support and PrEP adherence support from family and friends; accordingly labeled as ‘social support’. Factor loadings were very similar between the complete case (n = 315) and the TSML sensitivity analysis (n = 371). The Cronbach’s alpha coefficients for the overall HPRM scale and the three subscales indicated good to excellent internal consistency: overall HPRM scale was 0.92; ‘self-efficacy’ was 0.90; ‘PrEP disclosure’ was 0.71; and ‘social support’ was 0.80.

### Predictive validity of the HPRM

Among the 371 young women who accepted PrEP, 25% (n = 92) had high adherence at month three. Of those who had high adherence at month three, 64% (n = 59) had persistent high adherence at both months three and six. The median of the overall HPRM scale and the three subscales (self-efficacy, PrEP disclosure, and social support) scores were generally high: the median of the overall HPRM score was 3.26 (IQR: 2.91, 3.61); ‘self-efficacy’ was 3.38 (IQR: 3.00, 3.69), ‘PrEP disclosure’ was 3.00 (IQR: 2.50, 3.5); and ‘social support’ was 3.00 (IQR: 3.00, 4.00). The overall scale and the three subscales significantly predicted higher PrEP adherence (Table 3), for each unit increase in the average score when DBS TFV-DP concentration was treated as a continuous outcome measure: overall HPRM scale,103 fmol/punch (95% CI: 16, 189); ‘self-efficacy’, 90 fmol/punch (95% CI: 7, 172) fmol/punch; ‘PrEP disclosure’, 68 fmol/punch (95% CI: 19, 117 fmol/punch) and ‘social support’ 58 fmol/punch (95% CI: 2, 113 fmol/punch). The overall HPRM scale, and all subscales were suggestive of higher odds of PrEP adherence at month three. ‘PrEP disclosure’ was marginally associated with higher odds of PrEP adherence (OR 1.36, 95% CI: 1.00 to 1.86). Although the HPRM scale and all three factors suggest odds of persistently high adherence at months three and six, only ‘PrEP disclosure’ was significantly predictive of persistent high adherence (OR 1.50, 95% CI: 1.03 to 2.21).

**Table 3:** Overall HPRM scale and the subscales scores as predictors of PrEP adherence at three months and persistent adherence at months three and six.

	DBS concentration ^a^, ^b^		Odds of high adherence ^c^,^b^		Odds of persistent high adherence ^c^, ^d^	
	fmol/punch (95% CI)	P-value	OR (95% CI)	P-value	OR (95% CI)	P-value
**HPRM overall score (N = 315)**	102.88 (16.19, 189.22)	0.02	1.26 (0.74, 2.15)	0.40	1.47 (0.76, 2.85)	0.25
**Self-efficacy (N = 324)**	89.59 (6.72, 172.46)	0.03	1.26 (0.75, 2.12)	0.39	1.30 (0.69, 2.47)	0.42
**PrEP disclosure (N = 350)**	68.34 (19.36, 117.32)	0.01	1.36 (1.00, 1.86)	0.05	1.50 (1.03, 2.21)	0.04
**Social support (N = 361)**	57.92 (2.01, 113.75)	0.04	1.14 (0.81, 1.62)	0.45	1.30 (0.85, 1.99)	0.23

Covariate: site

^a^ DBS TFV-DP concentration as a continuous outcome

^b^ Month three DBS TFV-DP

^c^ High adherence: DBS TFV-DP ≥ 700 fmol/punch

^d^ Persistent high adherence at months three and six

There was no association between perception of HIV risk and increase in overall HPRM scale (0.01, 95% CI: -0.10, 0.12) or subscales: ‘self-efficacy’ (0.02, 95% CI:-0.10, 0.14), ‘PrEP disclosure’ (-0.05, 95% CI: -0.23, 0.14), and ‘social support’ (0.03,-0.13, 0.19).

### Comparison of baseline and month three HPRM scores

Month three HPRM scores were similar to baseline scores: median of the month three overall HPRM score was 3.22 (IQR: 2.95, 3.59); the ‘self-efficacy’ subscale was 3.31 (IQR: 2.69, 3.69), the ‘PrEP disclosure’ subscale was 3.25 (IQR: 2.75, 3.75); and the ‘social support’ subscale was 3.00 (IQR: 3.00, 4.00). The intraclass correlations between the baseline and month three scales were high: the overall HPRM scale was 0.99; ‘self-efficacy’ was 0.98; ‘PrEP disclosure’ was 0.92, and ‘social support’ was 0.83. For each unit increase in month three overall HPRM scale (OR: 108.25, 95% CI: 4.05, 212.00), and subscales ‘self-efficacy’ (OR: 103.85, 95% CI: 20.21, 187.49), and ‘PrEP disclosure’ (OR: 91.99, 95% CI: 9.16, 174.82) were significantly predictive of month six adherence when DBS TFV-DP concentration was treated as a continuous outcome measure. The ‘PrEP disclosure’ subscale at month three (OR 1.67, 95% CI: 1.13, 2.45) predicted the odds of high PrEP adherence at month six. The overall HPRM scale (OR 1.88, 95% CI: 1.02, to 3.48) and subscales ‘self-efficacy’ (OR 1.38, 95% CI: 0.75, 2.52) and ‘social support’ (OR 1.35, 95% CI: 0.88, 2.05) were also associated with a nonsignificant higher odds of PrEP adherence at month six.

## Discussion

In a study of South African and Zimbabwean AGYW in an open-label PrEP demonstration project, a measure to assess HIV prevention readiness at enrollment was associated with intracellular TFV-DP levels, an objective measure of PrEP adherence. Exploratory factor analysis identified three subscales ‘self-efficacy’, ‘PrEP disclosure’, and ‘social support’, which were predictive of increased DBS TFV-DP concentration at month three. The ‘PrEP disclosure’ subscale predicted the odds of having persistently high PrEP adherence (DBS TFV-DP ≥700 fmol/punch) at months three and six. In addition, the month three ‘PrEP disclosure’ subscale predicted the odds of high adherence at month six. The average month three overall scale and subscales ‘self-efficacy’ and ‘PrEP disclosure’ also predicted an average half-dose PrEP per week for each unit increase in the score, based on TFV-DP 200 fmol/punch being associated with one dose per week [33]. These findings indicate that providers could use full or brief HPRM subscales (e.g., ‘PrEP disclosure’ subscale) to determine HIV prevention readiness. Where potential PrEP users have low scores, this can prompt additional counseling at PrEP initiation and adherence support after PrEP initiation to mitigate the challenges to taking PrEP pills consistently.

Our study’s three subscales of HPRM differed from those for the HIV treatment readiness measure (HTRM). HTRM is a validated tool to assess HIV *treatment* readiness of adolescent men and women who live with HIV in the U.S. In contrast, HPRM assessed the HIV *prevention* readiness of AGYW in South Africa and Zimbabwe. Unlike the HTRM, where exploratory factor analysis identified two factors, *medication beliefs* and *connection with care* (i.e., provider characteristics), with the HPRM, these factors loaded under a single ‘self-efficacy’ factor along with housing stability items. In HTRM, disclosure of living with HIV and social support items loaded into a single subscale labeled *disclosure* and did not predict future viral suppression. With the HPRM, ‘PrEP disclosure” and social support’ were standalone subscales, and each predicted future higher PrEP adherence with higher scores. There are multiple reasons for the differences between HTRM and HPRM subscales. Unlike in the U.S., access to PrEP was primarily available through research sites in southern Africa during the implementation of the HPTN 082 study. Community awareness of PrEP and the belief in PrEP effectiveness were low, which may explain why medication belief and provider characteristics loaded into the subscale ‘self-efficacy’ for HPRM [34–36]. Due to societal norms, concerns about disclosing PrEP use could have differed by site and likely were conditional on perceived general support from partners, family, and friends. For some AGYW, PrEP disclosure could have been difficult if they were concerned about stigma associated with PrEP pills being mistaken for HIV treatment [11, 36, 37]. They might also have feared that disclosure would lead to the perception of promiscuity by their partners or family, which could threaten housing stability [11, 16, 34, 38].

There are several limitations to this study. First, although the comparison with month three scores suggests temporal stability, it may be fitting to administer HPRM at screening and enrollment (within 30 days) to establish test-retest reliability. Second, this study was limited to AGYW from South Africa and Zimbabwe. Other representative samples from sub-Saharan Africa with high HIV incidence should be considered in a validation study to increase the generalizability of the HPRM. Likewise, validation of the HPRM in other adolescent and young populations where PrEP use is low, such as U.S. adolescents, may be valuable [39, 40]. Lastly, we could not assess if the HPRM was associated with PrEP acceptance because 95% of the AGYW in HPTN 082 initiated PrEP [10]. It will be useful to examine what underlying factors are related to PrEP acceptance in future work.

## Conclusions

The findings of this study that the HPRM overall scale and the three subscales individually demonstrated good reliability (i.e., internal consistency) among African AGYW in youth-friendly clinic settings in southern Africa are encouraging. The ‘PrEP disclosure’ subscale, as a brief tool, has the potential to help identify AGYW who may need help with disclosing their PrEP use to supportive adults. It appears that the HPRM administered three months after starting PrEP can be used to identify young women whose adherence may be declining and may need adherence support. The HPRM has potential utility for routine use in other populations as a low-cost tool with low administrative burden to assess HIV prevention readiness in settings where PrEP adherence is a challenge for adolescents and young people. Future work will assess the replicability of the ‘self-efficacy’ and ‘social-support’ subscales after item revision, such as refining, deleting, or adding questions. Additionally, after modifying the items to be culturally appropriate, assessment of the validity should be conducted in among populations where HIV incidence is high and PrEP use is low.

## Supporting information

S1 Appendix HPRM questionnaire. (PDF)
