# Supplementary material for: Development of a tool to assess HIV prevention readiness of adolescent girls and young women in HPTN 082 study
Source: PLoS One. 2023 Feb 24;18(2):e0281728. doi: 10.1371/journal.pone.0281728 (PMC9956790; doi:10.1371/journal.pone.0281728)
Supplement: S1 Appendix — (PDF) [file pone.0281728.s001.pdf]

HIV PREVENTION READINESS MEASURE (HPRM)

Screening/Baseline Version

1. I am ready to start taking medication (PrEP) to protect against HIV.
   1=Strongly Disagree
   2=Disagree
   3=Neither Agree nor Disagree
   4=Agree
   5=Strongly Agree

2. I will tell most of the people that I live with that I am taking PrEP.
   1=Strongly Disagree
   2=Disagree
   3=Neither Agree nor Disagree
   4=Agree
   5=Strongly Agree

3. I believe taking PrEP can keep me healthy.
   1=Strongly Disagree
   2=Disagree
   3=Neither Agree nor Disagree
   4=Agree
   5=Strongly Agree

4. Taking PrEP would give me bad side effects. (R)
   1=Strongly Disagree
   2=Disagree
   3=Neither Agree nor Disagree
   4=Agree
   5=Strongly Agree

5. I would know how to contact the study staff if I had problems or questions about the PrEP medication.
   1=Strongly Disagree
   2=Disagree
   3=Neither Agree nor Disagree
   4=Agree
   5=Strongly Agree

6. I feel like I have a stable place to live.
   1=Strongly Disagree
   2=Disagree
   3=Neither Agree nor Disagree
   4=Agree
   5=Strongly Agree

7. If I don't take my PrEP medication exactly as instructed, I might get infected with HIV.
   1=Strongly Disagree
   2=Disagree

3=Neither Agree nor Disagree
4=Agree
5=Strongly Agree

8. I have a strong, trusting relationship with the study staff.
   1=Strongly Disagree
   2=Disagree
   3=Neither Agree nor Disagree
   4=Agree
   5=Strongly Agree

9. I would know who and when to call for refills of my PrEP medication.
   1=Strongly Disagree
   2=Disagree
   3=Neither Agree nor Disagree
   4=Agree
   5=Strongly Agree

10. Sometimes a homeless shelter is the only place I have to sleep. (R)
    1=Strongly Disagree
    2=Disagree
    3=Neither Agree nor Disagree
    4=Agree
    5=Strongly Agree

11. I will tell most of my family and friends that I am taking PrEP.
    1=Strongly Disagree
    2=Disagree
    3=Neither Agree nor Disagree
    4=Agree
    5=Strongly Agree

12. Taking PrEP medication would not really help me. (R)
    1=Strongly Disagree
    2=Disagree
    3=Neither Agree nor Disagree
    4=Agree
    5=Strongly Agree

13. Even when it may be difficult, I will be able to tell the study staff if I miss doses of my PrEP medication.
    1=Strongly Disagree
    2=Disagree
    3=Neither Agree nor Disagree
    4=Agree
    5=Strongly Agree

14. I regularly go to a clinic to seek advice about my health.
    1=Strongly Disagree
    2=Disagree
    3=Neither Agree nor Disagree
    4=Agree
    5=Strongly Agree

15. PrEP medication would be poison to my body. (R)
    1=Strongly Disagree
    2=Disagree
    3=Neither Agree nor Disagree
    4=Agree
    5=Strongly Agree

16. I want to start taking PrEP medications to protect against HIV infection.
    1=Strongly Disagree
    2=Disagree
    3=Neither Agree nor Disagree
    4=Agree
    5=Strongly Agree

17. My household members who I will tell that I am on PrEP will help me remember to take my medication.
    1=Strongly Disagree
    2=Disagree
    3=Neither Agree nor Disagree
    4=Agree
    5=Strongly Agree

18. Taking my PrEP medication as prescribed would keep me from getting sick.
    1=Strongly Disagree
    2=Disagree
    3=Neither Agree nor Disagree
    4=Agree
    5=Strongly Agree

19. I feel supported by my family and friends when times are tough.
    1=Strongly Disagree
    2=Disagree
    3=Neither Agree nor Disagree
    4=Agree
    5=Strongly Agree

20. I would take my PrEP medications even if they made me sick at first because the side effects would go away.
    1=Strongly Disagree
    2=Disagree
    3=Neither Agree nor Disagree
    4=Agree
    5=Strongly Agree

21. My family and friends that I will tell I am on PrEP would help me remember to take my medication.
    1=Strongly Disagree
    2=Disagree
    3=Neither Agree nor Disagree
    4=Agree
    5=Strongly Agree

22. I know that I will be able to take my PrEP medication correctly.
    1=Strongly Disagree
    2=Disagree
    3=Neither Agree nor Disagree
    4=Agree
    5=Strongly Agree

23. It would be important to me to take my PrEP medication correctly and on time every day.
    1=Strongly Disagree
    2=Disagree
    3=Neither Agree nor Disagree
    4=Agree
    5=Strongly Agree

24. I will not tell the people I live with that I am taking PrEP. (R)
    1=Strongly Disagree
    2=Disagree
    3=Neither Agree nor Disagree
    4=Agree
    5=Strongly Agree

25. How many people that you live will you tell that you are taking PrEP?
    1=No one
    2=Only one person
    3=Some people
    4=Most people
    5=Everyone

(R) denotes reverse-scored items

Follow-Up Version

1. I am ready to take medication (PrEP) to protect against HIV.
    1=Strongly Disagree
    2=Disagree
    3=Neither Agree nor Disagree
    4=Agree
    5=Strongly Agree

2. Most of the people I live with know that I am taking PrEP.
    1=Strongly Disagree
    2=Disagree
    3=Neither Agree nor Disagree
    4=Agree
    5=Strongly Agree

3. I believe taking PrEP can keep me healthy.
    1=Strongly Disagree
    2=Disagree
    3=Neither Agree nor Disagree
    4=Agree
    5=Strongly Agree

4. Taking PrEP gives me bad side effects. (R)
   1=Strongly Disagree
   2=Disagree
   3=Neither Agree nor Disagree
   4=Agree
   5=Strongly Agree

5. I know how to contact the study staff if I have problems or questions about the PrEP medication.
   1=Strongly Disagree
   2=Disagree
   3=Neither Agree nor Disagree
   4=Agree
   5=Strongly Agree

6. I feel like I have a stable place to live.
   1=Strongly Disagree
   2=Disagree
   3=Neither Agree nor Disagree
   4=Agree
   5=Strongly Agree

7. If I don't take my PrEP medication exactly as instructed, I might get infected with HIV.
   1=Strongly Disagree
   2=Disagree
   3=Neither Agree nor Disagree
   4=Agree
   5=Strongly Agree

8. I have a strong, trusting relationship with the study staff.
   1=Strongly Disagree
   2=Disagree
   3=Neither Agree nor Disagree
   4=Agree
   5=Strongly Agree

9. I know who and when to call for refills of my PrEP medication.
   1=Strongly Disagree
   2=Disagree
   3=Neither Agree nor Disagree
   4=Agree
   5=Strongly Agree

10. Sometimes a homeless shelter is the only place I have to sleep. (R)
    1=Strongly Disagree
    2=Disagree
    3=Neither Agree nor Disagree
    4=Agree
    5=Strongly Agree

11. Most of my family and friends know I am taking PrEP.
    1=Strongly Disagree

2=Disagree
3=Neither Agree nor Disagree
4=Agree
5=Strongly Agree

12. Taking PrEP medication does not really help me. (R)
    1=Strongly Disagree
    2=Disagree
    3=Neither Agree nor Disagree
    4=Agree
    5=Strongly Agree

13. Even when it is difficult, I let the study staff know if I miss doses of my PrEP medication.
    1=Strongly Disagree
    2=Disagree
    3=Neither Agree nor Disagree
    4=Agree
    5=Strongly Agree

14. I regularly go to a clinic to seek advice about my health.
    1=Strongly Disagree
    2=Disagree
    3=Neither Agree nor Disagree
    4=Agree
    5=Strongly Agree

15. PrEP medication is poison to my body. (R)
    1=Strongly Disagree
    2=Disagree
    3=Neither Agree nor Disagree
    4=Agree
    5=Strongly Agree

16. I want to take PrEP medications to protect against HIV infection.
    1=Strongly Disagree
    2=Disagree
    3=Neither Agree nor Disagree
    4=Agree
    5=Strongly Agree

17. My household members who know I am on PrEP help me remember to take my medication.
    1=Strongly Disagree
    2=Disagree
    3=Neither Agree nor Disagree
    4=Agree
    5=Strongly Agree

18. Taking my PrEP medication as prescribed keeps me from getting sick.
    1=Strongly Disagree
    2=Disagree
    3=Neither Agree nor Disagree
    4=Agree
    5=Strongly Agree

19. I feel supported by my family and friends when times are tough.
    1=Strongly Disagree
    2=Disagree
    3=Neither Agree nor Disagree
    4=Agree
    5=Strongly Agree

20. I take my PrEP medications even when they make me sick because the side effects go away.
    1=Strongly Disagree
    2=Disagree
    3=Neither Agree nor Disagree
    4=Agree
    5=Strongly Agree

21. My family and friends who know I am on PrEP help me remember to take my medication.
    1=Strongly Disagree
    2=Disagree
    3=Neither Agree nor Disagree
    4=Agree
    5=Strongly Agree

22. I know that I take my PrEP medication correctly.
    1=Strongly Disagree
    2=Disagree
    3=Neither Agree nor Disagree
    4=Agree
    5=Strongly Agree

23. It is important to me to take my PrEP medication correctly and on time every day.
    1=Strongly Disagree
    2=Disagree
    3=Neither Agree nor Disagree
    4=Agree
    5=Strongly Agree

24. I don't tell the people I live with that I am taking PrEP. (R)
    1=Strongly Disagree
    2=Disagree
    3=Neither Agree nor Disagree
    4=Agree
    5=Strongly Agree

25. How many people that you live with have you told that you are taking PrEP?
    1=No one
    2=Only one person
    3=Some people
    4=Most people
    5=Everyone

(R) denotes reverse-scored items
